# LGALS3BP is associated with selective cargo enrichment in neuroblastoma extracellular vesicles

**DOI:** 10.3389/fonc.2026.1966526

**Published:** 2026-10-15

**Authors:** Ilaria Cela, Fani Konstantinidou, Laura Amodei, Arianna Mercatelli, Francesca Piermatteo, Sara Santoro, Asia Pece, Maria Clara Staropoli, Alessia Lamolinara, Emily Capone, Valentina Gatta, Gianluca Sala

**Affiliations:** 1 Department of Innovative Technologies in Medicine and Dentistry, “G. d’Annunzio” University of Chieti-Pescara, Chieti, Italy; 2 Center for Advanced Studies and Technology (CAST), “G. d’Annunzio” University of Chieti-Pescara, Chieti, Italy; 3 Department of Neuroscience, Imaging and Clinical Sciences, School of Medicine and Health Sciences, “G. d’Annunzio” University of Chieti-Pescara, Chieti, Italy; 4 Unit of Molecular Genetics, Center for Advanced Studies and Technology (CAST), “G. d’Annunzio” University of Chieti-Pescara, Chieti, Italy; 5 Department of Science, “G. d’Annunzio” University of Chieti-Pescara, Chieti, Italy

**Keywords:** extracellular vesicles, LGALS3BP, neuroblastoma tumor microenvironment, miRNA cargo enrichment, SRSF1

## Abstract

**Aim:**

LGALS3BP (also known as Galectin-3-binding protein, Gal-3BP, or 90 K) is a multifunctional secreted glycoprotein involved in tumor progression and has emerged as a potential biomarker and therapeutic target in several malignancies, including neuroblastoma (NB). Increasing evidence suggests that LGALS3BP contributes to shaping the NB tumor microenvironment (TME), particularly through its enrichment on tumor-derived extracellular vesicles (EVs), where it may regulate tumor-stroma interplay. We investigated whether LGALS3BP contributes to stromal cell modulation in NB and is associated with changes in EV cargo composition.

**Methods:**

A patient-derived NB cell line naturally lacking LGALS3BP (hNB CTRL) and a matched counterpart ectopically re-expressing LGALS3BP (hNB LGALS3BP) were used. Bone marrow-derived mesenchymal stem cells (BMMSCs) were exposed to NB-derived conditioned media (CM) to assess cytokine secretion and LGALS3BP detection in recipient cells. EV cargo was analyzed by microRNA (miRNA) and proteomic profiling. Selected candidates were validated by qPCR and immunoblotting on NB-derived EVs.

**Results:**

LGALS3BP-containing CM was associated with an altered BMMSCs secretory profile, while tumor-derived extracellular LGALS3BP was detected in BMMSCs. Seven miRNAs were enriched in LGALS3BP-EVs and reduced in donor cells; four were increased in BMMSCs following EV exposure. Proteomic analysis revealed enrichment of RNA-binding proteins, including SRSF1, in LGALS3BP-EVs.

**Conclusions:**

LGALS3BP is associated with altered EV cargo composition and BMMSCs secretory responses, potentially contributing to tumor-stroma communication within the NB TME.

## Introduction

Neuroblastoma (NB) is the most common extracranial solid tumor in children and accounts for a significant proportion of pediatric cancer-related deaths. A hallmark of high-risk NB is its aggressive metastatic behavior and the presence of a highly immunosuppressive and poorly infiltrated tumor microenvironment (TME), often described as immunologically “cold” (1). This immune evasion contributes to the limited efficacy of current immunotherapeutic strategies. Extracellular vesicles (EVs) have emerged as critical mediators of tumor-host interactions, capable of modulating the behavior of surrounding stromal and immune cells. In NB, tumor-derived EVs play an active role in promoting tumor growth and metastatic spread, in part by reshaping the TME into a tumor-permissive niche (2, 3). LGALS3BP (also known as Galectin-3-binding protein, Gal-3BP, or 90 K) has garnered attention due to its multifunctional roles in immune modulation, cell adhesion, and tumor progression (4). LGALS3BP is a secreted, heavily glycosylated protein that is frequently overexpressed in several cancers, including NB (5–11). Our group recently demonstrated that LGALS3BP represents a promising disease biomarker and therapeutic target in NB (10). In particular, we found that LGALS3BP is highly enriched on the surface of cancer-derived EVs (5, 6, 8, 10), and that elevated levels of EV-associated LGALS3BP correlate with increased metastatic potential in preclinical NB models (5, 10). Moreover, we showed that targeting secreted LGALS3BP with an antibody-drug conjugate (ADC), namely 1959-sss/DM4, not only enhances lymphocyte infiltration into the tumor microenvironment but also synergizes with anti–PD-1 therapy in syngeneic preclinical NB models, resulting in improved tumor control (10).

In recent years, numerous studies have highlighted the ability of EVs to deliver tumor-associated specific EV cargo – including oncogenic proteins and miRNAs – that reprogram recipient cells in the TME, often promoting immunosuppression and tumor progression (3, 12–14). In this regard, it has recently shown that the presence of LGALS3BP is necessary for the efficient internalization of EVs containing immunosuppressive miRNAs (15). Furthermore, NB-derived EVs were capable of inducing mesenchymal stem cells (MSCs) to secrete pro-tumorigenic cytokines such as IL-6 in a LGALS3BP-dependent manner, highlighting its role in shaping the stromal landscape (16, 17).

To further investigate the potential contribution of secreted LGALS3BP, we ectopically reintroduced LGALS3BP into a patient-derived NB cell line (hNB) lacking endogenous expression of this protein (5, 18). As a representative model of the NB TME, bone marrow-derived mesenchymal stem cells (BMMSCs) were employed. EVs isolated from hNB cells expressing or lacking LGALS3BP were subjected to integrated miRNA profiling and proteomic analyses. These approaches identified a subset of miRNAs and proteins selectively enriched in LGALS3BP-associated EVs. Notably, the splicing factor SRSF1 – recently implicated in the regulation of miRNA loading into EVs (19) – was found to be enriched in LGALS3BP-positive EVs. Collectively, these findings support an association between LGALS3BP in NB-derived EVs and the selective enrichment of specific regulatory EV cargo, potentially contributing to stromal cell modulation within the NB microenvironment.

## Materials and methods

### Cell cultures and reagents

The primary human neuroblastoma hNB cell line was isolated from a non-MYCN amplified tumor metastasized in the neck of a 3-year-old male patient. The 11q and ALK status is not known (20). Human neuroblastoma SK-N-AS (RRID: CVCL_1700) cell line was purchased from American Type Culture Collection (ATCC; Rockville, MD, USA). Stable transduced hNB CTRL and LGALS3BP were grown in Roswell Park Memorial Institute (RPMI) 1640 (Gibco) under continuous puromycin selection (1 µg/mL), while SK-N-AS in Dulbecco’s modified Eagle’s medium (DMEM, Gibco; Whaltman, MA, USA). All media were supplemented with 10% heat-inactivated fetal bovine serum (FBS) (Gibco), 100U/mL penicillin and 100µg/mL streptomycin (Sigma-Aldrich Corporation, St. Louis, MO, USA). Bone marrow-derived mesenchymal stem cells (BMMSCs) were purchased from Sigma-Aldrich (#C-12974) and maintained in the Mesenchymal Stem Cell Expansion Medium (1x) (Sigma-Aldrich Corporation, #SCM015) supplemented with 4 ng/mL human FGF (Sigma-Aldrich Corporation; #F0291) and grown on plates coated with 0.1% gelatin. All cells were maintained at 37 °C in a humified air with 5% CO_2_. All cell lines were tested for mycoplasma contamination through polymerase chain reaction (PCR).

### Stable lentiviral transduction

hNB CTRL and hNB LGALS3BP cell lines were obtained by stable lentiviral transduction starting from hNB parental cell line, as previously described (10, 18). Briefly, lentivirus particles were produced by transient co-transfection of packaging cell line HEK 293T with a three-plasmid expression system using Lipofectamine 2000 (Invitrogen, Thermo-Fisher, Waltham, MA, USA). Lentiviral plasmid pLenti-CMV-PURO-hLGALS3BP for stable overexpression of human LGALS3BP cDNA was obtained as previously described (10, 18). HEK 293T cells were incubated for 6h with transfection reagents. Lentiviral particles were collected after 48h and 72h, and filtered through 0.45 µm pore sterile filters (Corning, NY, USA). The day before first cycle of transduction, 1.5 x 10^5^ hNB cells were plated. After 24 h cells were incubated for 6 hours with media containing viral particles together with 4 µg/mL of polybrene to increase transduction efficiency. After the first cycle of transduction, cells were washed and cultured with fresh medium overnight before a second cycle of transduction the day after. Cells were then let grow under standard growth conditions for 72h before being put under selection with 1 µg/mL of puromycin (Sigma-Aldrich). Selection was maintained throughout all experiments performed. LGALS3BP levels were assessed through quantitative real-time PCR (qRT-PCR) (18), western blotting and ELISA analysis.

### Extracellular vesicles isolation and characterization from cell culture supernatants

Once reached the 80% confluence, neuroblastoma cells were grown for 48 h in serum-free medium or in medium supplemented with EVs-depleted serum. Around 100 mL of cell culture supernatant was collected, and differential ultracentrifugation was performed for EVs isolation. Briefly, supernatants were centrifuged at 2000g and 10000g for 20 and 40 min at 4 °C to remove dead cells and cellular debris, and large extracellular vesicles, respectively. Then, to collect small extracellular vesicles, supernatants were ultracentrifuged at 100000g for 70 min at 4 °C and then washed with PBS and re-collected by centrifugation at 100000g for 70 min at 4 °C. Protein content was quantified by Bradford assay (Biorad) prior to western blotting analysis, miRNA and proteomic analysis, or functional assays. EVs were aliquoted and stored at -80 °C until their use.

### Western blotting analysis

Cell lysates were obtained from lysing cells in RIPA buffer (50mM Tris/HCl pH 7.6, 150mM NaCl, 1% NP-40, 0.5% Na-Deoxycholate, 0.1% SDS, 1mM EDTA pH 8) containing protease inhibitor cocktail (Sigma-Aldrich), phosphatase inhibitor cocktail (Roche, Thermo-Fisher), and Na_3_VO_4_ (Sigma-Aldrich) for 30 min at 4 °C. Lysates were clarified by centrifugation at 13000 rpm for 30 min at 4 °C. For western blotting analysis, equal amounts of protein lysates (20 µg), cell culture supernatants (25 µl) or EVs (2 µg), previously heated at 95 °C for 5 min, were subjected to SDS-PAGE electrophoresis and then electrotransferred to nitrocellulose membranes for Western blot analysis. Membranes were blocked for 1 h at RT with 5% non-fat dry milk in PBS with 0.1% Tween20. Membranes were then incubated overnight at 4 °C with the following primary antibodies: anti-human LGALS3BP (goat polyclonal; 1:1000, #AF2226; R&D Systems, MN, USA); anti-β-actin (mouse monoclonal; 1:40000, #A5441; Sigma-Aldrich); anti-Tsg101 (mouse monoclonal; 1:1000, #MA1-23296; Invitrogen); anti-CD9 (mouse monoclonal; 1:200, sc-59140; Santa Cruz Biotechnology, USA), anti-Calnexin (rabbit polyclonal; 1:1000, #ab22595; Abcam); anti-SRSF1 (mouse monoclonal; 1:500, #32-4500, Thermo Fisher). After three washes in PBS-0.1% Tween20, the membranes were hybridized with horseradish peroxidase (HRP)-conjugated secondary antibodies (goat, rabbit or mouse; Biorad, CA, USA). Detection of signal bands was performed with Clarity Western ECL substrate (#1705061; Biorad) or with Clarity Max Western ECL substrate (#1705062; Biorad). Images of membranes were acquired with a Uvitec ATOM reader (Cambridge, UK) and analyzed with Alliance Uvitec software (Cambridge, UK).

### Functional assays with BMMSCs

For conditioned media (CM) incubation, CM were obtained by plating 2 x 10^6^ neuroblastoma cells in 100mm Petri dishes; after 24 h, cells were grown in serum free medium that was collected after 48h. 1 x 10^5^ BMMSCs were seeded in 6-multiwell plates and starved in serum free medium for 24 h. CM derived from hNB CTRL (CM CTRL) or from hNB LGALS3BP (CM LGALS3BP) cells were added to BMMSCs for 24 h in the presence or not of 10 µg/mL of anti-LGALS3BP 1959 antibody. BMMSCs were then lysed and cell supernatants collected and stored at -80 °C for further analysis. For EVs incubation experiments, BMMSCs were incubated or not overnight with 10 µg of EVs. Cells were then harvested and collected for further analysis.

### RNA extraction

Total RNA, including microRNAs, was manually isolated from hNB CTRL and hNB LGALS3BP cell lines, as well as from BMMSCs (baseline), BMMSCs exposed to hNB CTRL EVs, BMMSCs exposed to LGALS3BP EVs, and from BMMSCs exposed to SK-N-AS EVs, by the Nucleospin miRNA kit (Macherey-Nagel, Milan, Italy) according to the manufacturer’s instructions. For hNB CTRL- and hNB LGALS3BP-derived EVs, RNA was extracted using the Total Exosome RNA and Protein Isolation Kit (Invitrogen, Thermo Fisher Scientific, Waltham, MA, United States). Quantity and quality of total RNA were assessed by microvolume UV–vis spectrophotometer NanoPhotometer (Implen, GmbH, Munich, Germany).

### MicroRNA TaqMan expression profiling

MicroRNA expression profiling in the parental hNB LGALS3BP compared to the hNB CTRL cell line and the hNB LGALS3BP-isolated EVs compared to the hNB CTRL-EVs was carried out by TaqMan™ Array Human MicroRNA A Cards v2.0 (Cat. 4398965, Applied Biosystems, Foster City, CA, United States) (21). For this initial screening, technical duplicate array cards were run for each condition using the same RNA sample respectively. A Megaplex RT Reaction was performed to reverse-transcribe 25 ng of miRNAs, using the Megaplex RT primer Pool A and TaqMan MicroRNA Reverse Transcription kit (Applied Biosystems, Waltham, MA). A preamplification reaction of complementary DNAs (cDNAs) was also executed through use of the TaqMan PreAmp Master Mix 2X and Megaplex PreAmp Primers pool A (Applied Biosystems, Waltham, MA). The preamplified products were then diluted with 0.1X TE at pH 8.0 and mixed with the TaqMan™ universal Master Mix II, no UNG (Applied Biosystems, Waltham, MA) and nuclease-free water. Finally, reactions were loaded into the TaqMan™ Array Human MicroRNA A plates and processed by qRT- PCR and the QuantStudio™ 7 Pro Real Time PCR detection system (Life Technologies, Carlsbad, CA, United States). Only miRNAs with Ct ≤ 35 and without outlier replicates were included in the analysis. In the TaqMan™Array Human MicroRNA A Cards, three TaqMan MicroRNA Assay endogenous controls were included to aid in data normalization and one TaqMan MicroRNA Assay not related to human was included as a negative control.

Raw data were analyzed by the Expression Suite software v1.3 (Thermo Fisher Scientific, Waltham, MA, USA). MicroRNAs were considered differentially expressed between groups when showing a fold change >1.4 or <0.7. Statistical significance of the miRNAs’expression in these conditions was assessed via two-tailed t-test, accepting p-values < 0.05. Due to the exploratory nature of this initial screening, no correction for multiple testing was applied. Candidate differentially expressed miRNAs were subsequently selected for formal validation via SYBR Green highly sensitive stem-loop RT-PCR.

### Validation and assessment of selected miRNAs by SYBR green highly sensitive stem-loop RT-PCR

Seven selected miRNAs, hsa-miR-26a-5p (MIMAT0000082; miRbase), hsa-miR-27b-3p (MIMAT0000419; miRbase), hsa-miR-29a-3p (MIMAT0000086; miRbase), hsa-miR-103a-3p (MIMAT0000101; miRbase), hsa-miR-152-3p (MIMAT0000438; miRbase), hsa-miR-340-5p (MIMAT0004692; miRbase) and hsa-miR-374b-5p (MIMAT0004955; miRbase), that were differentially expressed both in hNB LGALS3BP vs hNB CTRL cells and in LGALS3BP-EVs vs CTRL-EVs were subsequently validated by SYBR Green stem-loop RT-PCR. Validation was initially performed on the same RNA samples previously analyzed by TaqMan arrays (n = 2 per condition), as well as on two additional independent biological replicates (n = 2) derived from newly established cell cultures for each condition. For all samples, each RT-qPCR reaction was systematically executed in technical triplicates. MiRNAs were reverted from 25 ng of total RNA by high-capacity cDNA reverse transcription kit (Applied Biosystems, Foster City, CA, USA), using 1 μM of specific stem-loop reverse primers (**Supplementary Table 1**) as indicated by the following thermal protocol: 16 °C for 30 minutes, followed by 60 cycles at 30 °C for 30 seconds, 42 °C for 30 seconds and 50 °C for 1 second. A final incubation at 85 °C was also performed for 5 minutes in order to inactivate the reverse transcriptase. A target-specific pre-amplification reaction was carried out for each cDNA sample (22) to increase PCR sensitivity. Following pre-amplification, the amplification reaction for real-time PCR was carried out using the Maxima SYBR Green/ROX qPCR Master Mix (2X) (Thermo Fisher Scientific, Waltham, MA, USA) and PCR-specific 1 μM Forward Primer for each miRNA, as well as 1 μM of Universal Reverse Primer (**Supplementary Table 1**), under the following PCR conditions: 95 °C for 10 minutes, 40 cycles of 15 seconds at 95 °C and 40 seconds at 60 °C. Upon completion of the reaction cycles, melt curves were obtained by heating the reactions from 60 to 95 °C. MiRNA probe RNU6B was chosen as selected endogenous control. The specificity of the primers was confirmed by the presence of a single peak in the melt curve generated for all miRNAs.

To assess the expression of the selected miRNAs in BMMSCs after EV exposure, recipient BMMSCs were exposed to CTRL-EVs, LGALS3BP-EVs, SK-N-AS-EVs, or baseline conditions, and miRNA levels were measured using the same SYBR Green stem-loop RT-PCR protocol. For these assays, two technical runs were performed, with each individual reaction analyzed in technical triplicates for every target miRNA. Statistical analysis was performed as previously described (23).

### LC-MS/MS proteomic analysis of cellular lysates and EVs

EVs and corresponding cell lysates were obtained from hNB CTRL and hNB LGALS3BP cell lines. Cells were lysed in RIPA buffer, whereas EVs pellets were resuspended in PBS. Protein concentration for both cellular lysates and EV samples was determined using the Bradford assay (Bio-Rad Laboratories, Hercules, CA, USA). Protein samples (20 μg per sample) were digested using the Single-Pot Solid-Phase-Enhanced Sample Preparation (SP3) protocol following (24) by combining two types of carboxylate-functionalized hydrophilic and hydrophobic beads (1:1 ratio) (Sera-Mag SpeedBeads, GE Healthcare,Cytiva). Proteins were bound and subjected to overnight tryptic digestion at 37 °C. at a 1:10 enzyme-to-protein ratio. Peptide were analyzed in technical triplicate by nano-liquid chromatography-tandem mass spectrometry (nanoLC-MS/MS) using an UltiMate™ 3000 UPLC system coupled online to an Orbitrap Fusion™ Tribrid™ mass spectrometer (Thermo Fisher Scientific, Waltham, MA, USA). Chromatographic separation was performed on an EASY-Spray PepMap™ C18 column (75 μm ID×25 cm L, 2 μm particle size; Thermo Fisher Scientific) operated at a flow rate of 300 nL/min, with an injection volume of 3 μL per sample. Peptides were eluted using a linear gradient of solvent B (0.1% formic acid in acetonitrile) ranging from 5% to 90% over a total run time of 65 minutes. Raw LC-MS/MS data files were processed for label-free identification and quantification using Thermo Proteome Discoverer (PD) software version 2.4.0.305 (Thermo Fisher Scientific, Waltham, MA, USA) against the UniProt Homo sapiens database (Human_UP000005640, released February 2025). Search criteria specified trypsin as the cleavage enzyme, allowing up to 2 missed cleavages, with carbamidomethylation of cysteine (+57.021 Da) set as a fixed modification. Mass accuracy was maintained with a precursor mass tolerance set to 10 ppm. Contaminant proteins were filtered out, and robust quantification required a minimum of 1 unique peptide per protein. For comparative analysis between hNB control and hNB LGALS3BP samples (across both cellular lysates and EVs), protein abundances were normalized using the total peptide amount. Statistical evaluation was performed via ANOVA using unadjusted p-values within Proteome Discoverer version 2.4, and data visualizations, including Volcano plots and Heatmaps, were generated directly within the software environment. Proteins displaying an abundance ratio ≥ 2.0 or ≤ 0.5 and a statistical significance threshold of *p* < 0.05 were considered significantly regulated.

### EVs labeling, immunofluorescence and confocal imaging

After isolation, NB EVs were labeled with PKH67 using kit (#MINI67, Thermo Fisher) according to manufacturer’s instructions. 10 µg of PKH67-labeled EVs were incubated for 24 h with BMMSCs, plated on glass coverslips at a density of 1 x 10^5^. After incubation, cells were fixed with 4% paraformaldehyde for 10 min at RT and stained with AlexaFluor-546 conjugated phalloidin (#A22283; Invitrogen) and Hoechst3342 (1:500; Sigma-Aldrich) for 30 min at RT in the dark. Confocal images were acquired using a Zeiss LSM800 inverted confocal microscope system (Carl Zeiss, Germany).

### Enzyme-linked immunosorbent assays

Secreted human LGALS3BP levels were evaluated in cell culture supernatants by using an in-house sandwich ELISA developed as previously reported in (9). Briefly, NUNC Maxisorp 96-well plates (Thermo Fisher Scientific) were coated overnight at 4 °C with 2 µg/mL of anti-human LGALS3BP antibody 1959 (25). After blocking with 1% bovine serum albumin (BSA) in PBS-T 0.05% for 1 h at RT, 100 µl of each sample were added and incubated for 1 h a RT. After three washes with PBS-T 0.05%, plates were incubated with biotinylated anti-human LGALS3BP antibody 1959 (1.8 µg/mL) for 1 h at RT. For detection, after three washes with PBS-T 0.05%, Streptavidin-HRP was added (1:10000 dilution; Invitrogen, #SA10001) and incubated for 1 h at RT. After three washes, TMB ELISA Substrate Solution (#34021; Thermo Scientific) was added and the reaction was stopped with 2N H_2_SO_4_. Absorbance was measured at 450nm using a microplate reader (Tecan, Switzerland).

Secreted IL-6 was evaluated in cell culture supernatants by using human IL-6 uncoated ELISA kit (#88-7066-88; Thermo Fisher), while secreted VEGF by using Human VEGF DuoSet ELISA (#DY293B, R&D systems; USA) according to manufacturer’s instructions.

### Cytokine and chemokine quantification

A 32-plex screening panel (MilliplexR PLEXpedition Screening Panel #HPLX1-115SP-32M), was used to quantify 32 human cytokines and chemokines according to manufacturer’s instructions in cell culture media collected from BMMSCs exposed or not to CM from hNB CTRL or hNB LGALS3BP cells for 24 h. Analyte concentrations were measured using a Luminex 200™ system coupled with xPONENT^®^ software (Luminex Corp., USA). IL-3 and Perforin values were excluded from data presented since more than one experimental condition were out-of-range. Interpolated raw data were then transformed into z-scores.

### Bioinformatic analysis

Bioinformatic analysis of miRNA expression levels was performed by accessing the Gene Expression Omnibus (GEO) database. The GSE121513 dataset comprises RT-qPCR-based miRNA expression profiles from 117 samples, including 95 neuroblastoma tumors and 22 non-tumoral controls. The control group consisted of 8 embryonal stem cell lines, 7 fetal adrenal neuroblast samples, and 7 fetal adrenal cortex samples. The GSE16444 dataset contains miRNA expression profiles from 31 high-risk stage 4 neuroblastoma samples generated using the Agilent Human miRNA Microarray platform. All analyses were performed in R (version R 4.4.1) using Bioconductor packages including GEOquery, together with ggplot2 and reshape2 for data processing and visualization.

Bioinformatic analysis of SRSF1 expression was performed by accessing to R2 Genomics and data related to NB datasets (Kocak; SEQC) were retrieved. Analysis and images were obtained from R2 Genomics platform (https://r2platform.com).

### Statistical analysis

Statistical analysis performed and experimental sample numbers (n) are indicated in the figure legends. All statistical analysis was performed with GraphPad Prism 9.0 software and Proteome Discoverer version 2.4. A *p* < 0.05 was considered statistically significant.

## Results

To elucidate the potential contribution of LGALS3BP to tumor-stroma cross-talk modulation in neuroblastoma, we utilized a patient-derived NB cell line (hNB) previously described (5, 26). Notably, this cell line is characterized by a complete lack of LGALS3BP expression at both the mRNA and protein levels (5, 18). This unique feature provides a valuable experimental platform, as the stable reintroduction of LGALS3BP via lentiviral-mediated transduction enables the generation of an isogenic system suitable for direct comparative analyses. Using this approach, we generated LGALS3BP-expressing hNB cells, hereafter referred to as hNB LGALS3BP (18). These cells exhibited high intracellular expression and secretion of LGALS3BP at levels comparable to those observed in the neuroblastoma cell line SK-N-AS, which naturally expresses elevated amounts of LGALS3BP (**Figure 1A**). We further confirmed the abundant presence of the mature hyperglycosylated form of LGALS3BP in EVs released by hNB LGALS3BP, whereas no LGALS3BP was detected in parental hNB CTRL cells or their derived EVs (**Figure 1A**). Nanoparticle tracking analysis (NTA) showed that EV populations displayed the expected size distribution (**Supplementary Figure 1A**) and comparable number of EVs shed by both hNB CTRL and LGALS3BP cells (**Supplementary Figure 1B**). As a first step, we employed hNB cellular model to explore the potential effects of LGALS3BP-containing conditioned media (CM) on bone marrow-derived mesenchymal stem cells (BMMSCs). To this end, BMMSCs were exposed to CM derived from hNB cells lacking (CM CTRL) or expressing LGALS3BP (CM LGALS3BP). To assess whether exposure to LGALS3BP-containing CM could modulate the BMMSC secretory profile, we performed a multiplex Luminex analysis measuring 30 soluble mediators. BMMSCs exposed to CM LGALS3BP displayed broad changes in their secretome compared with untreated BMMSCs and BMMSCs exposed to CM CTRL, characterized by increased secretion of mediators associated with inflammatory stromal activation (IL-6, IL-8, IL-1α, IL-1β, IL-1Ra, and TNFα), myeloid-related factors (M-CSF, IL-10 and IL-23) and angiogenic mediators (VEGF-A), as well as innate inflammatory signaling-associated factors (i.e., IFNα2, IL-12 p40, IL-12 p70) (**Figure 1B**). To validate these findings, we measured by ELISA assay IL-6 and VEGF levels, as these pro-tumorigenic factors have been previously reported to be induced by LGALS3BP (16, 17, 25, 27). Notably, most the soluble factors analyzed were present at low or relatively low levels in the starting CM from both hNB CTRL and hNB LGALS3BP cells before being exposed to BMMSCs, as shown in the heatmap. Consistent with the Luminex analysis, BMMSCs exposed to CM LGALS3BP showed increased secretion of both IL-6 and VEGF, reaching statistical significance for VEGF only (**Figures 1C, D**). Importantly, these levels were specifically attributable to BMMSCs secretion, as both VEGF and IL-6 were virtually undetectable in NB-derived conditioned media. Collectively, these findings support a potential role for LGALS3BP in modulating the BMMSC secretory profile toward a more pro-tumorigenic state, potentially contributing to tumor-stroma communication within the TME. However, given the nature of the CM, it should be noted that both soluble and EV-associated forms of LGALS3BP are present. Interestingly, we detected LGALS3BP in BMMSCs following exposure to CM derived from hNB LGALS3BP cells, whereas this signal was not observed, or was markedly lower, in BMMSCs exposed to CM from hNB CTRL cells or in untreated BMMSCs. The detected band corresponded to the hyperglycosylated form of LGALS3BP (~100 kDa), consistent with the mature, secreted form of the protein. Importantly, the LGALS3BP signal detected in BMMSCs exposed to CM hNB LGALS3BP was markedly reduced upon addition of the anti-LGALS3BP therapeutic antibody 1959 (**Figure 1E**). These findings support an association of NB-derived extracellular LGALS3BP with BMMSCs following exposure to LGALS3BP-containing CM. To further examine the association of extracellular LGALS3BP with BMMSCs, BMMSCs were exposed to purified EVs isolated from hNB LGALS3BP or SK-N-AS cells. Notably, the hyperglycosylated form of LGALS3BP (~100 kDa) was detected in BMMSCs lysates following exposure to EVs from both LGALS3BP-positive cell models, whereas this band was not detected in untreated BMMSCs (**Supplementary Figure 2**). Together, these findings further support an association of extracellular, hyperglycosylated LGALS3BP with BMMSCs following exposure to purified LGALS3BP-positive EVs.

**Figure 1:**
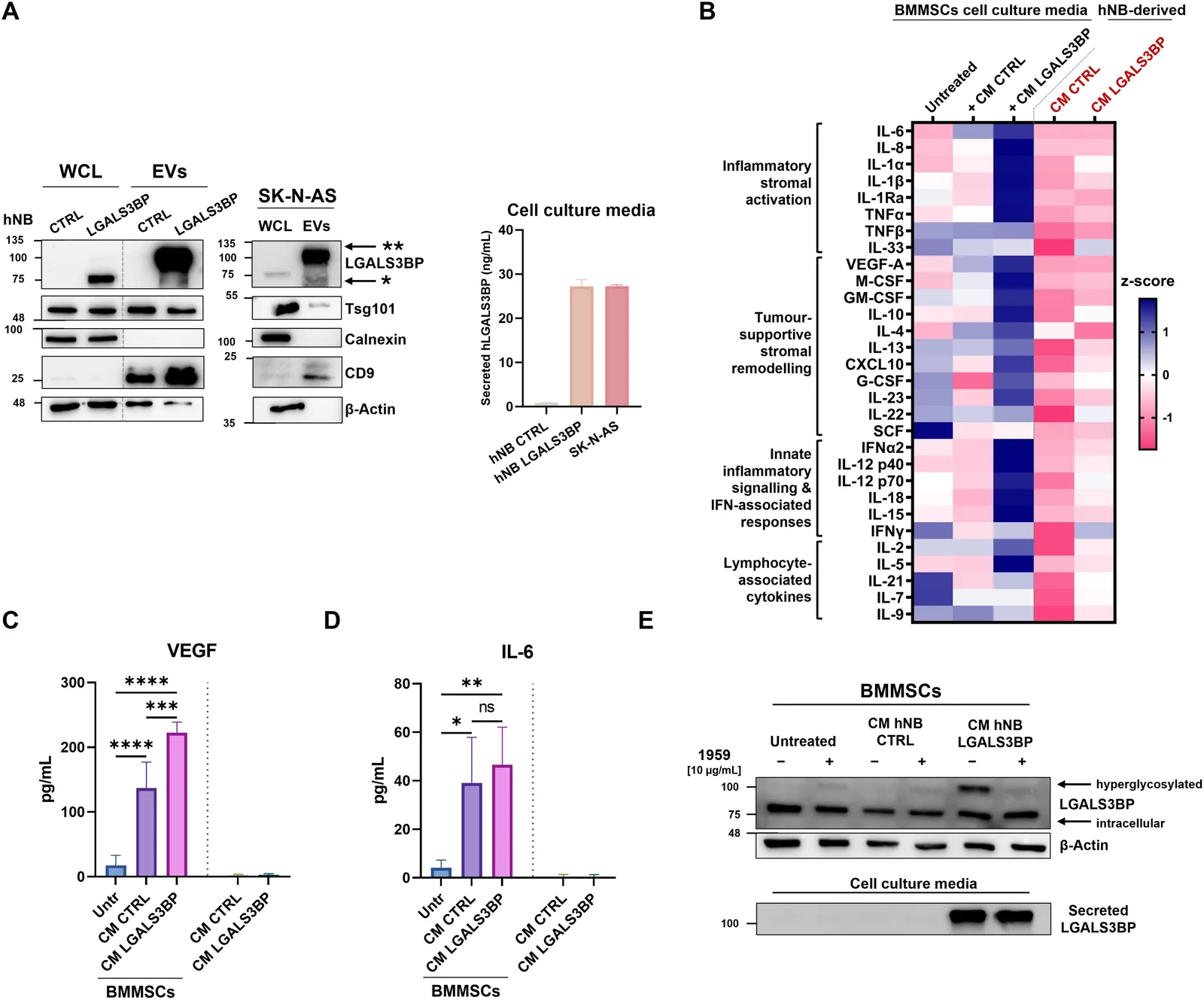
LGALS3BP induces the secretion of pro-tumorigenic factors and is associated with BMMSCs. **(A)** Western blot images of LGALS3BP protein levels in whole cell lysates (WCL) and in extracellular vesicles (EVs) from hNB CTRL, hNB LGALS3BP and SK-N-AS cells (left panel). Arrows indicate intracellular (*) and secreted hyperglycosylated (**) LGALS3BP protein. Positive and negative EVs markers are reported. Equal amounts of WCL (20 µg) and EVs (2 µg) were loaded per lane. Levels of LGALS3BP (ng/mL) secreted in cell culture media from hNB CTRL, hNB LGALS3BP, and SK-N-AS NB cells assessed through anti-human LGALS3BP 1959-based ELISA assay (right panel). Values are expressed as means ± standard deviation (SD). *n* = 2 (independent biological replicates). **(B)** Heatmap reporting secreted levels of 30 human cytokines, interferons and other soluble factors detected by multiplex Luminex analysis in the cell culture media of BMMSCs incubated or not with CM CTRL or CM LGALS3BP, and in the hNB-derived CM CTRL and CM LGALS3BP, all reported as z-score values. Color intensity represents the relative secretion level of each analyte, with increasing blue or coral intensity corresponding to higher or lower z-score values, respectively. Values were calculated based on *n=*3 independent biological replicates. **(C)** Secreted VEGF (*n* = 4, independent biological replicates) and **(D)** IL-6 (*n* = 3, independent biological replicates) levels (pg/mL) evaluated through ELISA assays in the supernatants from BMMSCs incubated or not with CM CTRL or CM LGALS3BP. Values are expressed as means ± SD. Statistical analysis was performed with one-way ANOVA test. *ns*, not significant, *p < 0.05, **p < 0.01, *** *p* < 0.001, **** *p* < 0.0001. **(E)** Western blot images of BMMSCs treated or not with conditioned media (CM) derived from hNB CTRL (CM hNB CTRL) or from hNB LGALS3BP cells (CM hNB LGALS3BP), in the presence or not of anti-LGALS3BP 1959 antibody (10 µg/mL) for 24 h. Secreted LGALS3BP levels in cell culture media from BMMSCs in corresponding conditions are reported. Upper arrow indicates hyperglycosylated LGALS3BP associated with BMMSCs, while lower arrow indicates BMMSCs intracellular LGALS3BP content. Equal amounts of protein lysates (20 µg) and cell culture media (25 µl) were loaded per lane. β-actin was used as loading control. *n* = 3, independent biological replicates.

After confirming a potential association of LGALS3BP in the modulation of BMMSCs secretome, and given that LGALS3BP is highly abundant on EVs, including those derived from NB cells (5, 10, 28), we sought to further investigate whether LGALS3BP expression is associated with changes in the molecular cargo of tumor-derived EVs. In this context, EV-associated LGALS3BP has been reported to enhance the delivery efficiency of EV-encapsulated miRNAs to recipient cells (15), prompting us to initially focus our characterization on EV miRNA content. EV-derived miRNAs are indeed known to contribute to the polarization of cells within the TME, including in NB (3, 12, 13). To deeply analyze the EVs miRNA cargo, we performed an exploratory human miRNA array screening to compare EVs isolated from parental and LGALS3BP-expressing hNB cells. Interestingly, we found a differential abundance in the expression of miRNAs between the two cell lines. In particular, among the 94 miRNAs profiled in EVs, 33 showed differential expression between LGALS3BP-EVs and CTRL-EVs, with a predominant upregulation in LGALS3BP-EVs (**Figure 2A**, left panel). In contrast, among the 118 miRNAs profiled in parental and LGALS3BP-expressing hNB cells, 29 showed differential expression, with a prevalent downregulation pattern in LGALS3BP-expressing cells compared to control (**Figure 2A**, right panel). From this exploratory screening, seven miRNAs were identified as to the EVs and cells comparison. These miRNAs were subsequently analyzed by stem-loop RT-PCR resulting at the same time significantly upregulated in EVs (**Figure 2B**, left panel; **Supplementary Figure 3**) and downregulated in cells (**Figure 2B**, right panel), suggesting a preferential enrichment of these miRNAs into LGALS3BP-EVs. To assess the interaction of NB-derived EVs with recipient cells within the TME, BMMSCs were used as cellular model. First, PKH67-labeled EVs were incubated with BMMSCs, followed by confocal imaging. The detection of PKH67-associated signal within BMMSCs indicated an interaction of NB-derived EVs with recipient cells (**Figure 2C**). Next, we investigated whether exposure to hNB-derived CTRL-EVs or LGALS3BP-EVs was associated with changes in miRNA abundances in recipient BMMSCs by analyzing miRNA expression levels. Interestingly, we found that four out of the seven miRNAs previously identified (miR-340-5p, miR-27b-3p, miR-103a-3p and miR-152-3p) were increased in BMMSCs exposed to LGALS3BP-EVs compared to BMMSCs exposed to CTRL-EVs or untreated BMMSCs, suggesting higher availability of LGALS3BP-associated miRNAs within recipient cells (**Figure 2D**). In contrast, miR-29a-3p, miR-374b-5p and miR-26a-5p did not show a similar increment pattern in BMMSCs exposed to LGALS3BP-EVs, despite their increased abundance in LGALS3BP-EVs relative to CTRL-EVs. This discrepancy may indicate that enrichment of a specific miRNA in EVs is not necessarily coupled to increased miRNA abundance in recipient cells, suggesting that additional factors may influence the fate of EV-associated miRNAs following exposure. To extend this approach to a neuroblastoma cell line naturally expressing high levels of LGALS3BP, BMMSCs were exposed to EVs derived from the endogenously high LGALS3BP-expressing SK-N-AS NB cell line. Interestingly, miRNA analysis revealed an upregulation of miR-340-5p, miR-27b-3p, and miR-103a-3p, but not miR-152-3p, in BMMSCs following SK-N-AS-EVs incubation (**Supplementary Figure 4**). These findings suggest that specific miRNAs enriched in LGALS3BP-associated EVs may be detected at increased levels in BMMSCs following EV exposure, although this pattern was not observed for all EV-enriched miRNAs. We next performed an *in silico* analysis of publicly available neuroblastoma (NB) datasets to compare the expression levels of selected miRNAs between normal tissues and NB samples, retrieving data for miR-27b, miR-340, and miR-152. As shown in **Figure 2E**, miR-27b and miR-340 were significantly upregulated in NB samples compared with normal controls, whereas miR-152 showed a more variable expression pattern without significant differences. To explore the potential relevance of these selected miRNAs, we performed a preliminary analysis of predicted targets of the LGALS3BP-EVs enriched miRNAs, highlighting candidates such as PPARG/PPARγ and DNMT1 implicated in metabolic and epigenetic regulation, respectively (**Supplementary Figure 5**) (29, 30).

**Figure 2:**
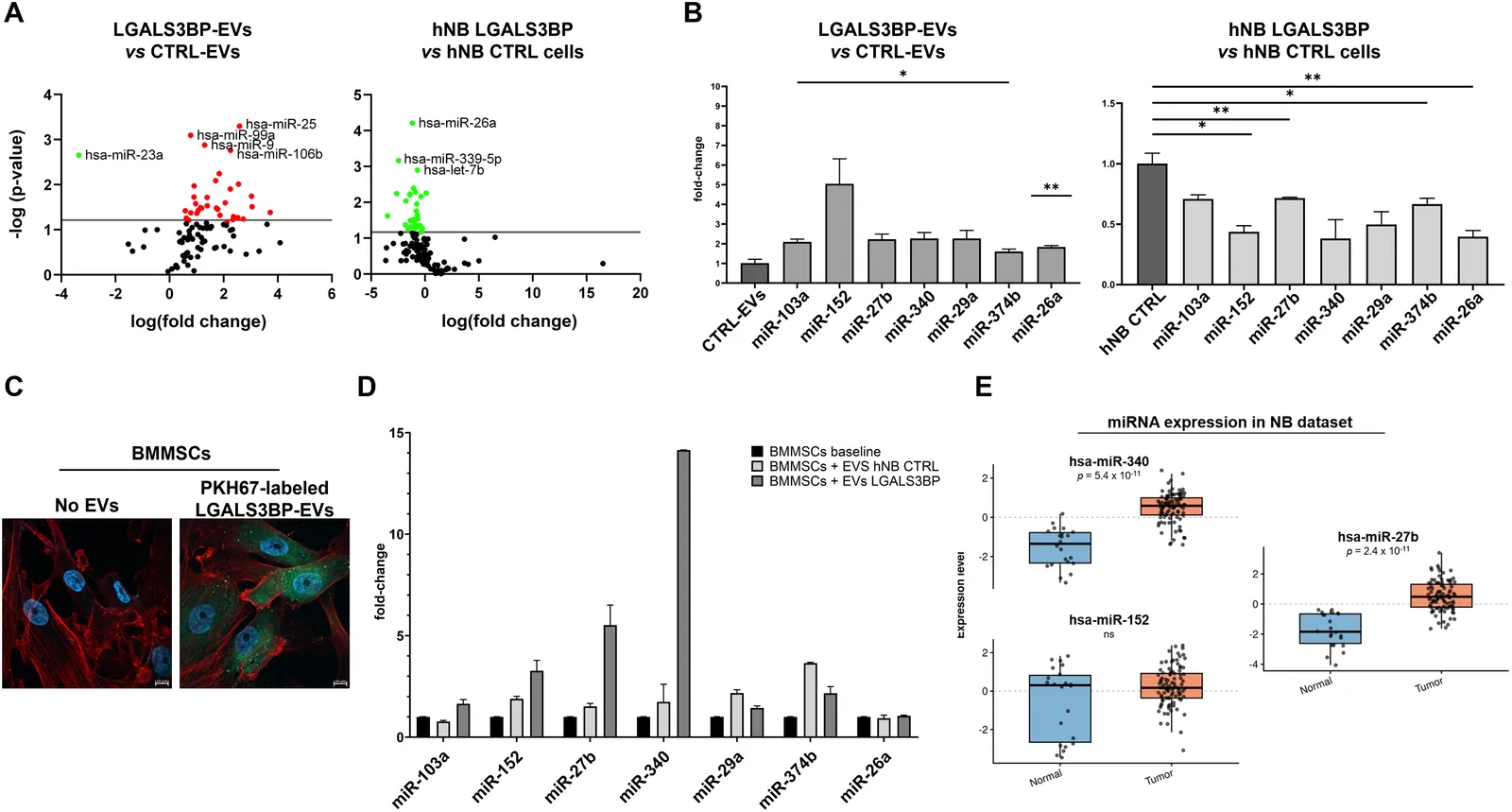
NB LGALS3BP-EVs show selective enrichment of specific miRNAs and increased miRNA abundance in recipient BMMSCs following EV exposure. **(A)** Volcano plots reporting differential expression levels of miRNAs detected in LGALS3BP-EVs compared to CTRL-EVs (left panel) or in parental hNB LGALS3BP cells compared to hNB CTRL cells (right panel), analyzed by TaqMan Array Human MicroRNA A Cards. Screening was performed in technical duplicates (*n* = 2 technical array cards per condition). miRNAs meeting the predefined thresholds based on differential expression and *p*-values (unadjusted two-tailed Student’s t-test) calculated from the technical replicates are reported as red dots (Fold Change cutoffs > 1.4; *p* < 0.05) for up-regulated miRNAs or green dots (Fold Change cutoffs < 0.7; *p* < 0.05) for down-regulated miRNAs. miRNAs with the highest differential expression are specifically labeled. **(B)** Expression levels of miRNAs evaluated by stem-loop RT-qPCR detected in LGALS3BP-EVs (left panel) or in hNB LGALS3BP cells (right panel). Values are reported as fold change of miRNA expression levels over CTRL-EVs (set at 1) or over hNB CTRL cells (set at 1), respectively. Data represent means ± SD from n = 2 independent biological replicates, with each qPCR reaction analyzed in technical triplicates. For each biological replicate, the mean of *n* = 3 technical replicates was calculated, and the resulting values were used to calculate the overall mean and SD shown in histograms. Statistical analysis was performed as reported in (23). *p < 0.05; **p < 0.01. **(C)** Confocal images of association of PKH67-labeled LGALS3BP-EVs with BMMSCs. Green dots correspond to PKH67-labeled LGALS3BP-EVs, red fibers correspond to actin cytoskeleton, while nuclei are stained with DAPI. Scale bar: 10µm. *n=*2, independent biological replicates. **(D)** Expression levels of miRNAs evaluated by stem-loop RT-qPCR detected in BMMSCs exposed to CTRL-EVs or to LGALS3BP-EVs. Values are reported as fold change of miRNA expression levels over untreated BMMSCs (baseline; set at 1). Data are presented as overall means ± SD calculated from the means of *n* = 2 technical runs, each performed in *n* = 3 technical replicates. Both technical runs were performed using the same *n* = 1 biological replicate. **(E)** Box plot showing expression levels of miR-27b-3p, miR-340-5p, and miR-152-3p measured by RT-qPCR in neuroblastoma tumors (n = 95) and normal controls (n = 22). Each dot represents an individual sample, and expression values were normalized prior to analysis. Student’s t-test was performed, and *p*-values are reported for each miRNA analyzed.

In a parallel approach, we further investigated the protein cargo specifically enriched in LGALS3BP-associated EVs by characterizing the proteomic content of hNB-derived EVs through shotgun proteomic analysis of isolated vesicles.

Consistent with the miRNome profiling, an exploratory proteomic screening identified a predominant increase in protein abundance in LGALS3BP-EVs compared with CTRL-EVs (**Figure 3A**). Notably, proteins showing increased abundance in LGALS3BP-EVs included several RNA-binding factors belonging to the Serine/Arginine-rich splicing factor (SRSF) family (**Figure 3B**). Among the candidate proteins identified in this exploratory screening, SRSF1 was validated by western blot analysis, supporting its increased abundance in LGALS3BP-EVs compared with CTRL-EVs, while no differences were observed in total cellular lysates (**Figure 3C**). It’s important to highlight that SRSF1 is an oncogenic splicing factor that has recently been implicated in the selective sorting of miRNAs into tumor-derived EVs (19). Moreover, analysis of EVs derived from SK-N-AS cell line confirmed vesicular SRSF1 expression concomitant with high levels LGALS3BP (**Figure 3C**). Interestingly, EV-associated SRSF1 displayed a different apparent molecular size compared with the intracellular form detected in whole-cell lysates (**Figure 3C**), suggesting that SRSF1 may undergo distinct post-translational modifications or proteolytic processing in association with EVs; however, the molecular basis of this difference remains to be determined.

**Figure 3:**
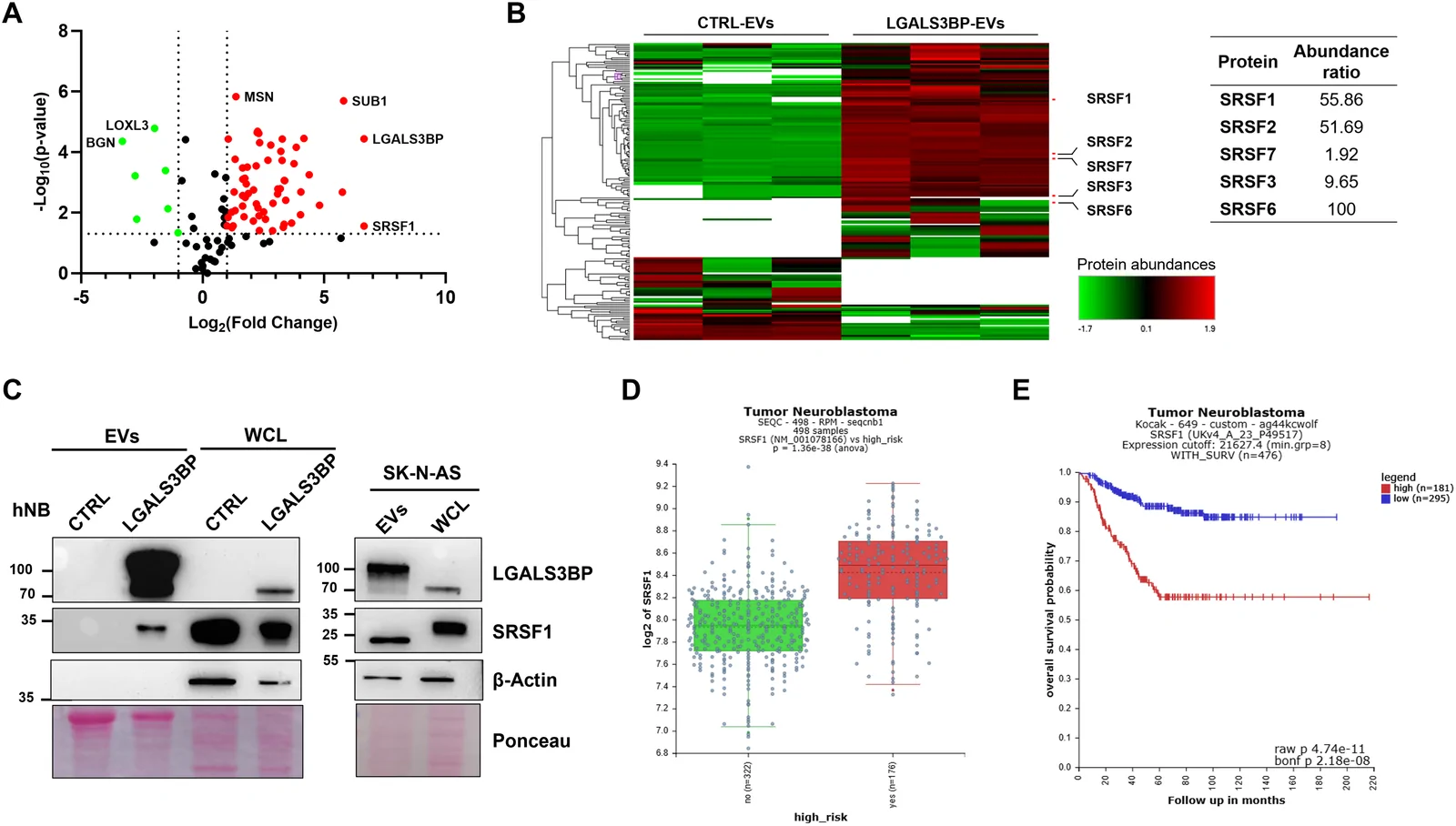
NB LGALS3BP-EVs show enrichment of specific RNA-binding proteins including the oncogenic splicing factor SRSF1. **(A)** Volcano plot reporting proteins showing differential abundance in LGALS3BP-EVs compared with CTRL-EVs from a single biological replicate (*n* = 1) analyzed in technical triplicate. Proteins meeting the predefined thresholds based on abundance ratio and *p*-values calculated from the technical triplicates are reported as red or green dots for up-regulated (abundance ratio ≥ 2.0, *p* < 0.05) or down-regulated (abundance ratio ≤ 0.5, *p* < 0.05) proteins, respectively. Statistical analysis was performed using one-way ANOVA within Proteome Discoverer v2.4, while GraphPad Prism 9.0 was used exclusively for graphical visualization. Proteins with the highest differential expression are specifically labeled. **(B)** Heatmap showing differentially expressed proteins identified in CTRL-EVs and LGALS3BP-EVs from a single biological experiment (*n* = 1) analyzed in technical triplicate. Each column represents an individual technical replicate for the indicated condition. Protein abundance is represented by a color scale ranging from green (decreased abundance) to red (increased abundance), standardized over a range of z-scores from −1.7 to +1.9. White cells indicate undetected proteins for which no abundance ratio could be calculated. Statistical significance referred to a single biological replicate (*n* = 1) analyzed in technical triplicate was evaluated using one-way ANOVA within Proteome Discoverer v2.4 (*p* < 0.05, absolute fold-change ≥ 2.0). SRSF family proteins are highlighted in the heatmap and listed in the corresponding table, together with their respective abundance ratios referred to a single biological replicate (*n* = 1) analyzed in technical triplicate. **(C)** Western blot images of LGALS3BP and SRSF1 protein levels in WCL and EVs of hNB CTRL, hNB LGALS3BP, and SK-N-AS cells. Equal amounts of WCL (20 µg) and EVs (8 µg) were loaded per lane. Ponceau is reported as loading control. *n=*2, independent biological replicates. **(D)** SRSF1 mRNA expression levels (log2) in NB patients classified as non–high-risk (no, n = 322) or high-risk (yes, n = 176) in the SEQC dataset. p = 1.36 × 10^−8^, ANOVA. **(E)** Kaplan–Meier overall survival analysis of NB patients stratified by low (n = 295) or high (n = 181) SRSF1 expression in the Kocak dataset. Raw *p* = 4.74 × 10^−11^; Bonferroni-adjusted *p* = 2.18 × 10^−8^. Datasets were accessed through the R2 Genomics Analysis and Visualization Platform (https://hgserver1.amc.nl/cgi-bin/r2/main.cgi).

Given the previously reported involvement of SRSF1 in miRNA sorting into tumor-derived EVs, we further investigated its clinical relevance in NB by interrogating publicly available patient datasets. Analysis of the SEQC cohort demonstrated significantly higher SRSF1 expression in high-risk compared with non–high-risk neuroblastoma patients (**Figure 3D**). Consistently, high SRSF1 expression was significantly associated with poorer overall survival compared with low SRSF1 expression (**Figure 3E**), as evaluated in the Kocak cohort. Collectively, these data associate elevated SRSF1 expression with aggressive disease features and unfavorable clinical outcome in neuroblastoma.

## Discussion

The ability of the primary tumor to remodel the tumor microenvironment is a key feature of many cancers. This process is mediated by the secretion of a wide range of factors, such as cytokines and growth factors, and, importantly, by the release of EVs, which play a central role in intercellular communication. In this context, LGALS3BP has emerged as one of the most abundant proteins associated with EVs, a feature that has been consistently reported across multiple cancer models, including neuroblastoma (NB) (5, 6, 8, 10, 28, 31). In addition, LGALS3BP was recently found to be upregulated in EVs derived from blood samples of NB patients compared to healthy controls (32). Notably, DeClerck and colleagues previously reported that LGALS3BP can induce IL-6 release from bone marrow-derived mesenchymal stem cells (BMMSCs) (16, 17). In the present study, we explored the potential contribution of LGALS3BP to the modulation of BMMSCs, a key stromal component of the NB TME, with a particular focus on its association with changes in EV cargo composition. To address this question, we took advantage of a cellular model based on a patient-derived neuroblastoma cell line (hNB) (20, 26). A distinctive feature of this cell line is the complete absence of LGALS3BP expression at both the mRNA and protein levels, as well as in the conditioned medium, indicating a lack of secretion (5). Using lentiviral-mediated gene delivery, we generated a stable hNB cell line expressing and secreting high amount of human LGALS3BP (18). This allowed us to directly compare conditioned medium from the same genetic background of neuroblastoma cells differing exclusively in LGALS3BP expression. By exposing BMMSCs to LGALS3BP-containing CM, multiplex Luminex profiling revealed an altered BMMSCs secretory profile, characterized by broad modulation of inflammatory mediators, angiogenic factors, and myeloid-related cytokines. Consistently, ELISA validation confirmed a significant increase VEGF secretion and an associated increase in IL-6 release by BMMSCs exposed to LGALS3BP-containing CM. These findings suggest that LGALS3BP may contribute to the modulation of specific pro-tumorigenic stromal responses rather than acting as the sole driver of cytokine induction. In particular, the LGALS3BP-associated increased release of VEGF, in line with previous observations (25, 27), is of particular interest given the established role of angiogenesis in NB progression and metastatic dissemination. Given the nature of the CM, both soluble and EVs-associated forms of LGALS3BP may be present; therefore, these findings do not allow us to discriminate between the effects potentially mediated by soluble LGALS3BP and those associated with EV-bound LGALS3BP.

Interestingly, the hyperglycosylated form of LGALS3BP (~100 kDa) was detected in BMMSCs following exposure to CM derived from LGALS3BP-expressing NB cells, and its signal was markedly reduced upon addition of the anti-LGALS3BP therapeutic antibody 1959. Notably, biochemical analyses performed using purified EVs further showed that the hyperglycosylated form of LGALS3BP could also be detected in BMMSC lysates following exposure to EVs derived from LGALS3BP-positive NB cell models. Collectively, these findings support an association of extracellular, tumor-derived LGALS3BP with BMMSCs following exposure to LGALS3BP-containing CM or purified LGALS3BP-positive EVs, while further studies will be required to define the precise cellular fate and localization of LGALS3BP in recipient cells as well as the functional consequences of this association.

Furthermore, through omics-based analyses, we characterized the miRNA and protein cargo of EVs isolated from control hNB and hNB LGALS3BP cells. This analysis identified seven miRNAs selectively enriched in LGALS3BP-containing vesicles and concomitantly reduced in LGALS3BP-expressing donor cells, consistent with the preferential enrichment of specific miRNAs in LGALS3BP-associated EVs (33, 34). Notably, four of these miRNAs were found increased in recipient BMMSCs following exposure to LGALS3BP-positive EVs, suggesting that their abundance may be altered in recipient cells following EV exposure. However, these findings remain exploratory and should be interpreted descriptively, requiring further validation to determine additional factors that might influence the fate of EV-associated miRNAs following uptake by recipient cells. Although the functional consequences of the association of these miRNAs with LGALS3BP-EVs remain to be directly investigated, *in silico* target analysis identified several potential targets associated with biological pathways that may be affected by the enrichment of these specific miRNAs in LGALS3BP-EVs, including PPARG/PPARγ and DNMT1. In line with our target prediction analyses, previous studies identified PPARγ as a validated target of miR-27b (35, 36). Given the central role of PPARγ in adipogenic differentiation and mesenchymal plasticity (37), it is possible that increased availability of miR-27b in BMMSCs following exposure to LGALS3BP-associated EVs could affect adipogenic differentiation programs and contribute to a more activated, tumor-supportive stromal phenotype. Interestingly, although miR-27b has been reported to exert tumor-suppressive effects in NB, its role appears highly context-dependent, with both oncogenic and tumor-suppressive activities described across different cancer settings (35, 36). Similarly, miR-340 and miR-103a, which were increased in BMMSCs exposed to LGALS3BP-EVs, have been associated with context-dependent tumor-suppressive or tumor-promoting functions in cancer (38–43). In particular, miR-340 and miR-103a have been validated to target PD-L1 (44) and PTEN (38), respectively, linking these miRNAs to pathways involved in immune modulation and tumor progression. Collectively, these findings raise the possibility that selective enrichment of specific miRNAs in EVs may contribute to tumor-stroma communication, while potentially altering the intracellular availability of these miRNAs in NB cells. However, whether these miRNAs effectively modulate their predicted targets and contribute to the observed stromal responses remains to be experimentally established.

The exploratory proteomic profiling further revealed changes in EV protein cargo associated with LGALS3BP expression. LGALS3BP-positive EVs were enriched in several RNA-binding proteins, including members of the serine/arginine-rich splicing factor (SRSF) family, among which SRSF1 was experimentally validated as showing increased abundance in LGALS3BP-EVs. Beyond its canonical function in alternative splicing, SRSF1 has emerged as a multifunctional regulator involved in RNA metabolism, translation, and cellular stress responses (45). Recent evidence has also linked SRSF1 to selective miRNA sorting into tumor-derived EVs (19, 46). In this context, SRSF1 enrichment in LGALS3BP-containing EVs identifies this RNA-binding protein as a potential candidate involved in the distinct vesicular miRNA profile observed in our model, although its contribution to EV cargo composition remains to be established.

Interestingly, SRSF1 has also been implicated in the regulation of angiogenic pathways in neuroblastoma, where it sustains VEGF165 expression and secretion downstream of the YAP/RUNX2 signaling axis (47). Although this function has been described in tumor cells, the known involvement of SRSF1 in both EV-mediated RNA regulation and VEGF-driven angiogenic programs supports its consideration as a potential contributor in the context of LGALS3BP-associated tumor-promoting mechanisms. However, whether SRSF1 functionally contributes to the biological effects associated with LGALS3BP and to tumor-stroma communication remains to be experimentally elucidated.

In addition, post-translational modifications of SRSF1, including arginine methylation, have been shown to influence its RNA-binding properties and consequently its interaction with specific miRNAs. In particular, SRSF1 demethylation reduces its affinity for selected miRNAs and promotes their enrichment within EVs (46), suggesting that not only SRSF1 abundance, but also its molecular status, may influence EV cargo selection. The clinical relevance of these observations is further supported by patient dataset analyses showing that elevated SRSF1 expression is associated with high-risk neuroblastoma and poorer overall survival. However, these clinical data refer to tumor-cell SRSF1 expression and do not specifically establish a role for EV-associated SRSF1. Together with our experimental observation of SRSF1 enrichment in LGALS3BP-associated EVs, these findings support the need to elucidate whether vesicular SRSF1 may contribute to EV-mediated tumor-stroma communication.

Taken together, our findings identify specific EV cargo associated with LGALS3BP, providing a basis for further investigation of the molecular mechanisms underlying tumor-stroma communication.

More broadly, tumor-derived EVs are increasingly recognized as important mediators of tumor-stroma communication within the TME, conveying selected and specific cargo to stromal and other TME recipient cells. Through their molecular cargo, EVs can modulate processes including stromal reprogramming, angiogenesis, immune responses, and tumor progression. Importantly, the composition of EV cargo can vary according to the cellular source and biological context, highlighting the relevance of selective cargo enrichment in shaping intercellular communication (3, 48–50).

Nonetheless, this study has limitations. First, although we observed selective miRNA enrichment in LGALS3BP-containing EVs and increased miRNA abundance in recipient BMMSCs, these data refer to one single experiment and therefore biological relevance should be further addressed. Whether LGALS3BP physically interacts with RNA-binding proteins such as SRSF1, alters endosomal trafficking, or modulates EV biogenesis pathways requires further investigation. Second, the biological impact of these miRNAs on BMMSCs phenotype was inferred from expression analyses and target prediction, but should be confirmed through functional studies. Finally, *in vivo* validation will be needed to assess the potential relevance of these LGALS3BP-associated observations in relation to metastatic niche formation and therapeutic resistance mechanisms.

In conclusion, our findings expand the current view of LGALS3BP in neuroblastoma by identifying an association between LGALS3BP expression and changes in EV cargo composition. Beyond its established enrichment in EVs and its proposed role in EV-mediated intercellular communication, LGALS3BP appears associated with the selective enrichment of regulatory miRNAs and RNA-binding proteins, including SRSF1, highlighting potential molecular features that may be relevant to EV-mediated communication with stromal cells. This LGALS3BP-associated EV cargo profile may contribute to the modulation of tumor-stroma communication within the NB TME and may provide new opportunities for biomarker development and therapeutic targeting.

## Conclusions

In conclusion, our study expands the current understanding of LGALS3BP as an emerging player in tumor-stroma crosstalk within the NB TME, identifying a previously unrecognized association between LGALS3BP and changes in EV cargo composition. Consistent with previous observations, our findings further support an association between LGALS3BP expression and the stromal secretory response, while also revealing selective enrichment of regulatory miRNAs and RNA-binding proteins within NB-derived EVs. Although exploratory, these findings provide new insights into the potential contribution of LGALS3BP to tumor-stroma communication in NB and support its further investigation as a potential therapeutic target and biomarker.

## Data Availability

The mass spectrometry proteomics data have been deposited to the ProteomeXchange Consortium via the PRIDE [52] partner repository with the dataset identifier PXD085150 and 10.6019/PXD085150; further inquiries can be directed to the corresponding author.
