# Supplementary material for: LGALS3BP is associated with selective cargo enrichment in neuroblastoma extracellular vesicles
Source: Front Oncol. 2026 Oct 15;16:1966526. doi: 10.3389/fonc.2026.1966526 (PMC13716570; doi:10.3389/fonc.2026.1966526)
Supplement: Supplementary file 1 [file SupplementaryFile1.docx]

**LGALS3BP is associated with selective cargo enrichment in Neuroblastoma Extracellular Vesicles**

Ilaria Cela^1,2^, Fani Konstantinidou^3,4^, Laura Amodei^1,2^, Arianna Mercatelli^1,2‡^, Francesca Piermatteo^1,2†^, Sara Santoro^1,2^, Asia Pece^1,2#^, Maria Clara Staropoli^1,2^, Alessia Lamolinara^1,3^, Emily Capone^2,5^, Valentina Gatta^3,4^, and Gianluca Sala^1,2^

*^1^Department of Innovative Technologies in Medicine & Dentistry, “G. d’Annunzio” University of Chieti-Pescara, Chieti, Italy;*
*^2^Center for Advanced Studies and Technology (CAST), “G. d’Annunzio” University of Chieti-Pescara, Chieti, Italy;*
*^3^ Department of Neuroscience, Imaging and Clinical Sciences, School of Medicine and Health Sciences, "G. d'Annunzio" University of Chieti-Pescara, Chieti, Italy; ^4^ Unit of Molecular Genetics, Center for Advanced Studies and Technology (CAST), “G. d’Annunzio” University of Chieti-Pescara, Chieti, Italy; ^5^ Department of Science, “G. d’Annunzio” University of Chieti-Pescara, Chieti, Italy.*

**Supplementary Methods**

**Characterization of NB EVs**

Size and concentration of EVs were characterized by nanoparticle tracking analysis (NTA) on a ZetaView system (Particle Metrix, Meerbusch, Germany) equipped with a blue laser (488 nm, 40 mW) and a long-pass emission filter. The autofocus was calibrated to ensure optimal particle visualization. Consistent and optimized post-acquisition settings were applied throughout the analyses. Each sample was analysed by capturing videos across 11 fields, with the resulting measurements compiled into reports detailing all relevant parameters, such as mean and median EV sizes.

Number of total EVs shed by NB cells was evaluated by flow cytometry. For the assay, 1 x 10^6^ hNB CTRL or LGALS3BP cells were plated. The day after, cells were put in serum-free medium for 24 h after which were collected to evaluate the total number of EVs as previously reported in (51). Parental cells were stained with 7AAD in order to count only the alive cells. Ratio were calculated by dividing the total number of EVs measured over the total number of alive parental cells.

**miRNA target prediction analysis**

Target genes of the selected miRNAs were identified using the R package multiMiR, which integrates experimentally validated and computationally predicted miRNA–mRNA interactions from multiple databases. Gene symbols were standardized according to HGNC nomenclature. Predicted interactions supported by at least two independent databases were retained and intersected with experimentally validated interactions to define a set of high-confidence targets. For each miRNA, the target genes with the highest level of supporting evidence were selected for downstream analyses and visualization.

**Supplementary Figures**

**Supplementary Figure S1. Characterization of CTRL-EVs and LGALS3BP-EVs from NB cells.** **(A)** Representative NTA profiles of EVs isolated from hNB CTRL (left panel) and hNB LGALS3BP cells (right panel). Diameter (nm) values are expressed in function of number of particles per mL. **(B)** Ratio of the total number of EVs shed by the same number of parental hNB CTRL or LGALS3BP cells. Values refers to means ± SD. *n*=2, independent biological replicates.

**Supplementary Figure S2. Hyperglycosylated LGALS3BP derived from NB-EVs is associated with BMMSCs.** Western blot images of BMMSCs incubated or not with EVs derived from hNB CTRL, hNB LGALS3BP, or SK-N-AS cells for 18 h. Secreted LGALS3BP levels in cell culture media from BMMSCs in corresponding conditions are reported. Upper arrow indicates hyperglycosylated LGALS3BP associated with BMMSCs, while lower arrow indicates BMMSCs intracellular LGALS3BP content. Equal amounts of protein lysates (20 µg), EVs (2 µg), and cell culture media (25 µl) were loaded per lane. Tsg101 is reported as EV marker. β-actin was used as loading control. n=2, independent biological replicates.

**Supplementary Figure S3. Enrichment of specific miRNAs in LGALS3BP-EVs compared to CTRL-EVs.** Expression levels of miRNAs evaluated by stem-loop RT-qPCR detected in LGALS3BP-EVs. Values are reported as fold change of miRNA expression levels over CTRL-EVs (set at 1). Values are expressed as means ± SD. Statistical analysis was performed as reported in ^[23]^. * p<0.05; ** p<0.01; *** *p*<0.001. *n*=2.

**Supplementary Figure S4. Increased abundance of selected miRNAs in BMMSCs following exposure to LGALS3BP-associated EVs derived from SK-N-AS NB cells.** Expression levels of miRNAs evaluated by stem-loop RT-qPCR detected in BMMSCs exposed to SK-N-AS-EVs and in SK-N-AS-EVs. Values are reported as fold change of miRNA expression levels over untreated BMMSCs (baseline; set at 1). Data are presented as overall means ± SD calculated from the means of *n*=2 technical runs, each performed in *n*=3 technical replicates. Both technical runs were performed using the same *n*=1 biological replicate.

**Supplementary Figure S5. Prediction of targets of selected miRNAs enriched in LGALS3BP-EVs.** Top 5 miRNA targets were obtained through multiMiR package by combining validated and predicted interactions for miR-27b-3p, miR-340-5p, miR-152-3p, and miR103a-3p. Predicted targets supported by at least two databases were retained and intersected with validated interactions to define a high-confidence target set. The five target genes with the highest level of support are shown for each miRNA. The y-axis represents the number of databases supporting each interaction.

**Supplementary Tables**

**Supplementary Table S1.** Stem-loop microRNA primer sequences.

| **microRNA** | **Primer sequences (5’–3’)** |
| --- | --- |
| RT_miR-26a | GTTGGCTCTGGTGCAGGGTCCGAGGTATTCGCACCAGAGCCAACAGCCTA |
| miR-26a_Forward | GCGGCGGTTCAAGTAATCCAG |
| RT_miR-27b | GTTGGCTCTGGTGCAGGGTCCGAGGTATTCGCACCAGAGCCAACGCAGAA |
| miR-27b_Forward | GCGGCGGTTCACAGTGGCTAA |
| RT_miR-29a | GTTGGCTCTGGTGCAGGGTCCGAGGTATTCGCACCAGAGCCAACTAACCG |
| miR-29a_Forward | GCGGCGGTAGCACCATCTGAA |
| RT_miR-152 | GTTGGCTCTGGTGCAGGGTCCGAGGTATTCGCACCAGAGCCAACCCAAGT |
| miR-152_Forward | GCGGCGGTCAGTGCATGACAG |
| RT_miR-103a | GTTGGCTCTGGTGCAGGGTCCGAGGTATTCGCACCAGAGCCAACTCATAG |
| miR-103a_Forward | GCGGCGGAGCAGCATTGTACA |
| RT_miR-340 | GTTGGCTCTGGTGCAGGGTCCGAGGTATTCGCACCAGAGCCAACAATCAG |
| miR-340_Forward | GCGGCGGTTATAAAGCAATGA |
| RT_miR-374b-5p | GTTGGCTCTGGTGCAGGGTCCGAGGTATTCGCACCAGAGCCAACCACTTA |
| miR-374b-5p_Forward | GCGGCGGATATAATACAACCT |
| RT-RNU6B | GTTGGCTCTGGTGAGGGTCCGAGGTATTCGCACCAGAGCCAACAAAAATATGG |
| RNU6B_Forward | GCGGCGGCGCAAGGATGACACG |
| Universal Reverse | GTGCAGGGTCCGAGGT |
